# Acute pharmacological effects of α-PVP in humans: a naturalistic observational study

**DOI:** 10.3389/fphar.2025.1626692

**Published:** 2025-07-02

**Authors:** Georgina De la Rosa, Esther Papaseit, Olga Hladun, Lourdes Poyatos, Dolly Andrea Caicedo, Martha Catalina Argote, Soraya Martín, Mireia Ventura, Nunzia La Maida, Annagiulia Di Trana, Silvia Graziano, Simona Pichini, Magí Farré, Clara Pérez-Mañá

**Affiliations:** ^1^ Clinical Pharmacology Department, Hospital Universitari Germans Trias i Pujol, Badalona, Spain; ^2^ Department of Pharmacology, Therapeutics and Toxicology, Universitat Autónoma de Barcelona, Barcelona, Spain; ^3^ Energy Control, Associació Benestar i Desenvolupament, Barcelona, Spain; ^4^ National Center on Addiction and Doping, National Institute of Health, Rome, Italy

**Keywords:** cathinones, alpha-pyrrolidinopentiophenone (α-PVP), pharmacology, new psychoactive substances, psychostimulants

## Abstract

**Introduction:**

Alpha-pyrrolidinopentiophenone (α-PVP) is a commonly consumed analogue of pyrovalerone, a synthetic cathinone with psychostimulant properties similar to those of 3,4-Methylenedioxypyrovalerone (MDPV) and cocaine. Since the pharmacology of α-PVP remains scarcely studied, we aimed to evaluate the acute pharmacological effects and its abuse potential in humans after intranasal administration.

**Methods:**

We carried out a non-controlled observational study in a naturalistic environment in nine participants (3 women and six men) with a previous history of psychostimulant use. Participants self-administered a single intranasal dose of 10mg or 20mg of α-PVP. The outcomes included physiological effects (systolic and diastolic blood pressure, heart rate, and temperature) and subjective effects (Evaluation of Subjective Effects of Substances with Abuse Potential questionnaire_VESSPA-SSE, the short form of the Addiction Research Center Inventory questionnaire_ARCI and visual analog scales_VASs) and were measured at different time points (0, 20 and 40 minutes and 1, 1.5, 2, 2.5, 3, 4 and 5 hours).

**Results:**

An acute increase in blood pressure and heart rate was observed that peaked 40 minutes after administration. Subjective effects also showed a rapid onset and disappeared 3 to 5 hours after administration.

**Discussion:**

α-PVP showed psychostimulant properties similar to those displayed by cocaine and empathogenic effects commonly associated with MDMA and other cathinones (eg. methylone) consumption.

## 1 Introduction

Alpha-pyrrolidinopentiophenone or α-PVP [alpha-pyrrolidinovalerophenone, 1-phenyl-2-(pyrrolidin-1-yl) pentan-1-one, flakka or gravel] is a molecular analogue of pyrovalerone. Both substances are synthetic cathinones, a group of among the most widely used new psychoactive substances (NPS) (Wood et al., 2016; Daziani et al., 2023). Synthetic cathinones were developed from cathinone, an alkaloid with psychostimulant properties present in the leaves of the shrub *Catha edulis* (Khat, qat, or cat) (Valente et al., 2014), to enhance the stimulant properties and modify the pharmacological effects of the parent compound, aiming to produce more potent or long-lasting effects while circumventing existing legal restrictions on natural cathinone derivatives (Banks et al., 2014). Once a synthetic cathinone entered the illicit market as “legal highs” or “legal euphorics” and was identified by law enforcement, it was swiftly banned by international law due to its potential for abuse. Consequently, new synthetic cathinones with modified structures were developed and introduced into the illicit market to evade legal controls while maintaining similar psychoactive effects. Although, most of these substances retain their primary activity as central nervous system stimulants, their chemical structure has evolved into different forms that modulate intensity, duration, and side effects (Daziani et al., 2023; Salomone et al., 2016).

α-PVP emerged in the NPS market as a legal alternative to 3,4- methylenedioxymethamphetamine (MDMA, midomafetamine), being widely consumed in the U.S. and other countries within the European Union (EU). Despite being known as a second-generation cathinone, it is the chemical precursor of 3,4-Methylenedioxypyrovalerone (MDPV), which belongs to the first generation of cathinones (Karila et al., 2018). With its renewed popularity, it began to be sold online, marketed as plant fertilizer, bath salt, insecticide, or labeled as a “research chemical” in the form of a crystalline white powder. Between 2011 and 2015, more than 200 acute intoxications and about 120 fatal intoxications associated with α-PVP were officially reported to the European Early Warning System by eight member States (European Monitoring Centre for Drugs and Drug Addiction, 2015). The appearance of several deaths linked to α-PVP consumption in the media led authorities to classify it as a prohibited substance temporarily in the U.S. in 2014 (definitely in 2017) and in the EU in 2015 (European Monitoring Centre for Drugs and Drug Addiction, 2015; Patocka et al., 2020; Drug enfocement administration US Department of Justice, 2017). Nevertheless, α-PVP continued to be detected in real cases of fatality, both alone or in combination with other drugs of abuse and NPS, especially in Finland and France (La et al., 2021). Similarly to other NPS, the prevalence of α-PVP should be considered underestimated due to the lack of pharmacological data to support the medical staff in the prompt diagnosis of related intoxications. Especially acute intoxications could be misinterpreted due to the unspecific symptoms which are often similar to other psychotropic substance intoxications (Taflaj et al., 2024).

α-PVP inhibits dopamine and norepinephrine reuptake through its transporters (DAT and NET), with a profile very similar to that of MDPV and cocaine (Meltzer et al., 2006; Marusich et al., 2014; Zwartsen et al., 2017; Rickli et al., 2015). However, it has a weaker inhibitory effect on the serotonin transporter (SERT) (Marusich et al., 2014). Evidence suggests that α-PVP’s selective blockade of catecholamine reuptake transporters may lead to a higher risk of addiction and adverse effects compared to non-selective substances, such as mephedrone or methylone (Marusich et al., 2014; Baumann et al., 2017). The desired effects of α-PVP include euphoria, increased sociability, heightened libido, enhanced perception, and increased energy. In addition to these, users report time distortion and paranoid delusions (Stanciu et al., 2017).

Regarding the dosage reported by consumers, intranasal doses of 1–5 mg are considered light, common from 5 to 10 mg, and strong from 15 to 25 mg. For oral route, doses of 5–10 mg (light), 10–25 mg (common), and 25–40 mg (strong) have been reported (Psychonautwiki, 2025). The minimum oral dose required to induce psychoactive effects is around 1–2 mg, whereas intense effects are reported with oral doses of 20–25 mg (Karila et al., 2018). Following intranasal administration, effects appear within minutes and last approximately 3 h, often requiring redosing within 30–120 min (leading to repeated or binge use). When taken orally, effects appear around 15 min post-ingestion and last up to 6 h (Stanciu et al., 2017). This difference may be due to more rapid entry of the drug when administered intranasally into the bloodstream producing fast effects and higher bioavailability like in the case of mephedrone (Papaseit et al., 2016). Some users recommend combining various administration routes to achieve faster and longer-lasting effects. The most frequently reported clinical manifestations of α-PVP intoxications in emergency rooms include tachycardia (92%), agitation (77%), hypertension (31%), hallucinations (38%), delirium (15%), and rhabdomyolysis (15%) (Beck et al., 2016; Umebachi et al., 2016). In some cases, hyperthermia, mydriasis, diaphoresis, seizures, and hypokalemia have also been observed (Beck et al., 2016). These physical symptoms, including hyperthermia and tachycardia, result from the sympathomimetic effects of α-PVP (Umebachi et al., 2016).

The primary causes of death linked to α-PVP use are cardiac infarction and pulmonary edema (Marinetti and Antonides, 2013; Sellors et al., 2014; Nagai et al., 2014; Sykute et al., 2015; Potocka-Banaś et al., 2017). Notably, cases of psychosis associated with high doses and sudden death have been reported (Khan et al., 2013; Eiden et al., 2013; Perez-Sagaseta de Ilurdoz et al., 2024) as well as a case of catatonia (Richman et al., 2018).

There is no published data on α-PVP abuse potential, acute physiological and subjective effects in humans, other than reports of acute intoxication and user-reported experiences on the internet. This limited information indicates that effects resemble those produced by other psychostimulants like MDPV (European Monitoring Centre for Drugs and Drug Addiction, 2016). Although concentrations and toxicity have been reported in case series of acute poisonings (Beck et al., 2016; Umebachi et al., 2016), recall bias and timing uncertainty make causality difficult to establish. On the other hand, conducting experimental studies involving illicit substances remains highly challenging due to the complex legal restrictions to obtain the substances and ethical considerations.

For these reasons we designed an observational study where data were collected prospectively with standardized evaluation tools including intensive assessments at the beginning to identify the peak values of the different outcomes. Moreover, the exact self-administered doses were known and adulteration of α-PVP and ingestion of other substances were previously discarded, avoiding polydrug use confounding. Furthermore, naturalistic studies like this one make it possible to observe the effects of drugs in settings where users typically consume these substances, thus providing data with greater ecological validity.

The choice of nasal insufflation (snorting) for the study was based on the fact that it is one of the most commonly used and reported route in cases of acute α-PVP poisoning with analytical confirmation (European Monitoring Centre for Drugs and Drug Addiction, 2015; Beck et al., 2016).

The present study aimed to assess the acute pharmacological effects of α-PVP following its administration in humans via the intranasal route in a naturalistic environment.

## 2 Materials and methods

### 2.1 Participants

The inclusion criteria for participants were as follows: individuals of any gender, aged between 18 and 45 years, healthy, with no history of psychiatric disorders, and with prior recreational use of psychostimulants and/or synthetic cathinones via the intranasal route. The exclusion criteria included: a history of significant medical or mental health disorders, such as substance use disorder (excluding nicotine), previous severe adverse reactions to psychostimulants, or being on long-term medication, and also pregnant women.

Participants were recruited through word-of-mouth in collaboration with the association Energy Control (Asociación Bienestar y Desarrollo, https://energycontrol.org/), a harm reduction organization that offers assessment and drug checking services. The study protocol received approval from the local Human Research Ethics Committee (CEIC HUGTiP, Badalona, Spain, ref. PI-18–267). All participants were fully informed about the study’s objectives and procedures and provided written informed consent before any study-related activities. The study was carried out in compliance with the Declaration of Helsinki and relevant Spanish research regulations (Biomedical Research Law 14/2007). Participants were financially compensated for their involvement in the study.

### 2.2 Study design

We carried out a non-controlled, prospective observational study in a naturalistic environment. The methodology, including the procedures and assessments, aligns with those used in our prior observational-naturalistic studies evaluating the acute effects of other NPS (Poyatos et al., 2021; Papaseit et al., 2021; Martínez et al., 2021; Papaseit et al., 2020a). Participants individually acquired the substance from unidentified suppliers, and Energy Control analyzed it. Powder of α-PVP underwent quali-quantitative analysis using a validated gas chromatography-mass spectrometry (GC/MS) method (Di Trana et al., 2025). The analysis confirmed that the α-PVP had a purity level greater than 95%, with no other toxic substances or adulterants detected. Participants self-administered α-PVP intranasal, selecting a dose based on their prior experience with the substance. Based on existing literature and user opinions, they could choose between two predetermined doses (10 mg or 20 mg). The doses were pre-weighed with a calibrated scale and deposited on a paper slip. The participants opened the paper, distributed its contents in two lines and proceeded to self-administration with a straw. The administration was completed in 1 minute and carried out in an intimate environment supervised by one of the researchers.

### 2.3 Procedures

Participants were asked to refrain from using any recreational drugs for 1 week prior to the selection visit and to avoid alcohol and caffeinated beverages for the 24 h leading up to the study session (day of administration). This period was considered necessary to avoid interactions as well as residual effects of other substances.

The selection visit was conducted 24–48 h before the study session. To confirm the participants’ eligibility, medical history (including mental health disorders), drug consumption history, and physical examination were carried out. Furthermore, they received detailed instructions and training on the procedures and questionnaires that would be used during the session.

The session took place in a private club, which was closed to the public, with participants arriving at 15:00 and remaining until 5 h after substance self-administration. Upon their arrival, urine samples were collected from each participant to screen for the presence of common drugs of abuse (including benzodiazepines, barbiturates, morphine, methadone, cocaine, amphetamines, methamphetamine, MDMA, THC, and tricyclic antidepressants) using the Drug-Screen Multi 10TD Test (Multi-Line, Nal Von Minden, Moers, Germany). Additionally, previous alcohol consumption was discarded with the assessment of breath alcohol concentrations (Drager Alcotest 5,820, Dragerwerk AG & Co., Lubeck, Germany). Urinary pregnancy test was performed in case of female participants (Glip Test Plus hCG Card^®^, Ref 30,701, Biosynex, Delemont, Switzerland) and must be negative to participate.

During the session, participants were free to engage in activities such as talking, reading, listening to music, or playing games except during the scheduled evaluation times. They were asked to refrain from discussing the effects of the substance among themselves.

Evaluations were conducted at baseline (before) and then at 20 min (0.33 h), 40 min (0.67 h), 1 h, 1.5 h, 2 h, 2.5 h, 3 h, 4 h, and 5 h following self-administration of α-PVP via the intranasal route. A light snack (piece of fruit) was provided 2 hours after administration. The assessments conducted at each time point followed this order: saliva sample (collected from −5 min to specific time point), vital signs (recorded from −5 min till the specific time point) and questionnaires (from the specific time point till 5 min after; first VASs, second ARCI, third VESSPA).

### 2.4 Physiological effects

Systolic blood pressure (SBP), diastolic blood pressure (DBP), and heart rate (HR) were measured using an automatic Omron monitor (Omron, Hoofddorp, Netherlands) with subjects seated at baseline, and at 20 min, 40 min, 1 h, 1.5 h, 2 h, 2.5 h, 3 h, 4 h, and 5 h after self-administration of α-PVP. The cutaneous temperature of the forehead was assessed using a contactless infrared thermometer (Beurer Ulm, Germany) at the same time points.

### 2.5 Subjective effects

Subjective effects were assessed at baseline, 20 min, 40 min, 1 h, 1.5 h, 2 h, 2.5 h, 3 h, 4 h, and 5 h after self-administration of α-PVP, utilizing a series of visual analog scales (VASs) and the Addiction Research Center Inventory 49 item-short form (ARCI) (same time points except 20 min). The Evaluation of Subjective Effects of Substances with Abuse Potential questionnaire (VESSPA-SSE) that was administered at baseline, 1 h, 2 h, 3 h, 4 h, and 5 h and the Positive and Negative Syndrome Scale for Schizophrenia (PANSS) at baseline and at 5 h.

VASs (100 mm, from “not at all” to “extremely”) were used to rate subjective effects as previously reported (Poyatos et al., 2021; Poyatos et al., 2022b).

The Spanish version of the 49-item short form of the ARCI is a standardized questionnaire with true/false responses validated to evaluate subjective effects of drugs of abuse (Lamas et al., 1994).

The VESSPA-SSE questionnaire measures changes in subjective effects caused by a number of drugs, mainly stimulants such as MDMA (Poyatos et al., 2021; Poyatos et al., 2022b).

The PANSS was used to evaluate psychotic symptoms (Poyatos et al., 2021; Poyatos et al., 2022b).

### 2.6 Oral fluid concentrations of α-PVP

Oral fluid (saliva) samples were collected using Salivette^®^ at baseline and at 20 min, 40 min, 1 h, 1.5 h, 2 h, 2.5 h, 3 h, 4 h, and 5 h following substance consumption. All samples were centrifuged after collection and stored at −20°C until analysis. After the liquid-liquid extraction at controlled pH with ethyl acetate of 50 μL OF; α-PVP and β-OH-α-PVP concentrations were respectively quantified with a gas chromatography-electronic ionization-tandem mass spectrometry method (GC-EI-MS/MS) and High-performance Liquid chromatography coupled to tandem high resolution mass spectrometry (HPLC-ESI-HRMS/MS) method, previously reported (Di Trana et al., 2025). The analytical methods were successfully validated for linearity, sensitivity, accuracy, precision, carryover, dilution integrity, matrix effect and recovery following a 5-day protocol, according to the OSAC for Forensic Sciences guidelines. All the parameters were within the acceptable criteria proposed by the above-mentioned guidelines. In particular, the GC-EI-MS/MS methods had a linear range between 50–1,000 ng/mL (LOD 10 ng/mL), while the HPLC-ESI-HRMS/MS method was linear between 1-300 ng/mL. All the methods proved to be sufficiently sensitive for the scope. One male subject was excluded in the reporting of concentrations due to outlier results in his oral fluid testing.

### 2.7 Statistical analysis

The determination of the sample size was based on the methodology of bioequivalence studies, which resulted in 8-9 subjects needed, considering an alpha risk of 0.05, a power of 80%, with a difference of at least 35% between 40 min values and baseline in the intensity/high effect and with 25% of variability.

Differences with respect to baseline were calculated for vital signs (SBP, DBP, HR and T) and subjective effects (VASs, ARCI and VESSPA). Maximum effects (Emax) and the time needed to reach maximum effects (Tmax) were also calculated for these outcomes. The areas under the curve of the effects (AUC) from 0 to 5 h using the trapezoidal rule were calculated for vital signs and subjective measures.

A two-way analysis of variance (ANOVA) was conducted to evaluate the influence of dose and gender on the different parameters calculated (Emax and AUC) of all vital signs and subjective effects. Given that any of the main effects showed significant differences, all participants were included in one group, independently of these factors. After that, we conducted a Dunnett post hoc test to compare the different time points with baseline values, which was adjusted for multiple comparisons.

A Correlation was conducted to evaluate the relationship between some subjective effects and oral fluid concentrations.

Statistical analysis was carried out using PASW Statistics version 18 (SPSS Inc., Chicago, IL, United States). Differences were considered statistically significant when the resulting p value was <0.05.

## 3 Results

### 3.1 Participants

A total of nine subjects (6 males and three females) participated in the study for self-administration of α-PVP. Participants had a mean age of 31.8 ± 5.9 years (range 24–42 years), weighed a mean of 68.6 ± 11.4 kg (range 48.3–79 kg) and had a mean body mass index (BMI) of 22.3 ± 3.6 kg/m2 (range 17.5–29.9 kg/m2). The self-administered intranasal dose of α-PVP was either 10 or 20 mg (average dose of 16.6 mg, corresponding to 0.25 mg/kg); five men and one woman received 20 mg, while two women and one man received 10 mg). All subjects were categorized as a single group for the results (as detailed in the statistical section).

All selected participants reported prior experience with psychostimulants, including NPS/synthetic cathinones, cocaine, MDMA, amphetamines, cannabis, and hallucinogens. Six were current tobacco smokers, and all of them reported consuming alcohol. Concerning psychostimulants (cathinones, cocaine, MDMA, amphetamines, methamphetamines, and 4-bromo-2,5-dimethoxyphenethylamine_2-CB) participants reported a median of 251 (range:66–1,156) lifetime uses, a median of 59 (range:26–254) uses in the past year and a median of 11 (range:1–40) uses in the past month. All subjects had negative urine drug tests at the start of the session. No clinical signs of intoxication were noted at baseline in any subject.

### 3.2 Physiological effects

**FIGURE 1:**
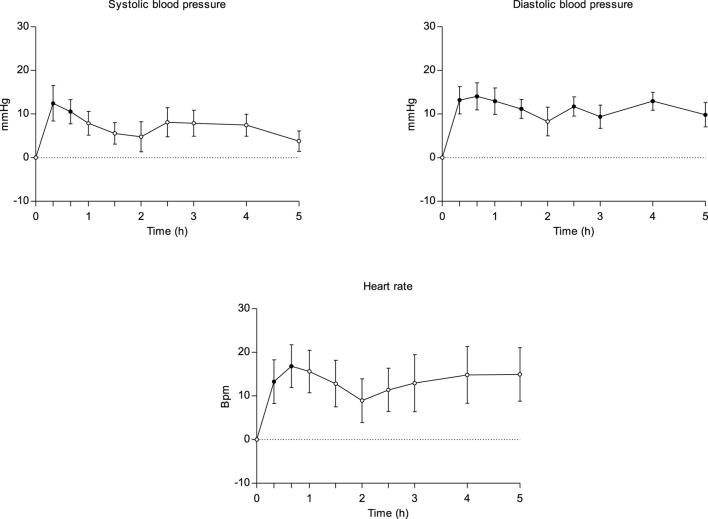
Time course (n = 9; mean ± standard error) of physiological effects (heart rate and blood pressure) following intranasal administration of 10–20 mg α-PVP. Significant differences from the baseline are indicated with filled symbols • (p < 0.05).

Table 1 summarizes the effects and parameters (peak effect_Emax, time to reach peak effect_Tmax, area under the curve from 0 till 5 h_AUC0-5h) of physiological outcomes following α-PVP self-administration. Furthermore, it includes statistically significant comparisons to baseline using the Dunnett test for the different time assessments. Additionally, Figure 1 shows the time course of heart rate and blood pressure.

**TABLE 1:** Summary of results on physiological measures.

Physiological effects	Parameters	Mean ± SD	Dunnett’s test
SBP (mmHg)	E_max_	148.4 ± 10.9	**a**, b
	T_max_ ^a^	0.66 (1.0−5.0)	
	AUC_0−5h_	688.76 ± 47.23	
DBP (mmHg)	E_max_	99.78 ± 10.00	**a, b, c, d, f, g, h, i**
	T_max_ ^a^	0.66 (1.0−5.0)	
	AUC_0–5h_	463.59 ± 44.22	
HR (bpm)	E_max_	98.78 ± 15.91	**a, b**
	T_max_ ^a^	0.66 (1.0−5.0)	
	AUC_0–5 h_	442.12 ± 76.76	
Temperature (°C)	E_max_	37.2 ± 0.07	c, **e, f, g, h, i**
	T_max_ ^a^	2.0 (1.0−5.0)	
	AUC_0−5h_	184.37 ± 0.35	

Emax, peak effects 0−5 h (differences from baseline) measured by mmHg (systolic blood pressure [SBP], diastolic blood pressure [DBP]), bpm (heart rate [HR]), °C (temperature [T]). A post-hoc Dunnett’s test for multiple comparisons was used. Statistical differences are presented as “a” p < 0.05, “**a**” p < 0.01 (times 0−0.33 h), “b” p < 0.05, “**b**” p < 0.01 (times 0−0.66 h), “c” p < 0.05, “**c**” p < 0.01 (times 0–1 h), “d” p < 0.05, “**d**” p < 0.01 (times 0–1.5 h), “e” p < 0 .05, “**e**” p < 0.01 (times 0−2 h) and “f” p < 0.05, “**f**” p < 0.01 (times 0−2.5 h), “g” p<0.05 “**g**” p < 0.01 (times 0−3 h), “h” p < 0.05, “**h**” p < 0.01 (times 0–4 h) and “i” p<0.05, “**i**” p<0.01 (times 0–5 h).

^a^ For T_max_ data are reported, as median and range.

α-PVP increased systolic blood pressure (SBP), diastolic blood pressure (DBP), and heart rate (HR). Maximum effects were +17.44 mmHg, +17.94 mmHg and +17.61 bpm, respectively. Compared to the baseline values, statistically significant differences were observed for SBP during the first hour, DBP from the first until the 5th hour, and HR the first hour. Conversely, there were no changes in body temperature.

### 3.3 Subjective effects

Overall, α-PVP produced moderate peak subjective effects on the different scales used. Effects began at 20 min, with maximum values occurring between 40 min and 1 h, and most effects nearly disappeared by 5 h. Table 2 presents the pharmacodynamic parameters for these outcomes.

**TABLE 2:** Summary of results on subjective effects.

Subjective effects	Parameters	Mean ±SD	Dunnett’s Test
*VAS intensity* (mm)	E_max_	39.5 ± 27.7	**a, b, c, d**
	T_max_ ^a^	0.66 (0.66−1.50)	
	AUC_0−5 h_	55.0 ± 56.7	
*VAS stimulated * (mm)	E_max_	43.2 ± 28.3	**a, b, c**, d
	T_max_ ^a^	0.66 (0.33−1.00)	
	AUC_0−5 h_	56.57 ± 56.88	
*VAS high* (mm)	E_max_	40.22 ± 29.35	**a, b, c**
	T_max_ ^a^	0.66 (0.33−1.50)	
	AUC_0−5 h_	54.59 ± 62.99	
*VAS good effects* (mm)	E_max_	43.67 ± 30.95	**a, b, c**, d
	T_max_ ^a^	0.66 (0.33−1.50)	
	AUC_0−5 h_	61.75 ± 64.71	
*VAS bad effects* (mm)	E_max_	1.00 ± 0.00	NS
	T_max_ ^a^	0.33 (0.00−2.00)	
	AUC_0−5 h_	1.09 ± 0.00	
*VAS liking* (mm)	E_max_	56.44 ± 37.33	**a, b, c**
	T_max_ ^a^	0.66 (0.33−1.50)	
	AUC_0−5 h_	85.17 ± 74.29	
*VAS clarity* (mm)	E_max_	54.11 ± 39.37	**a, b, c**, d
	T_max_ ^a^	0.66 (0.33−1.50)	
	AUC_0−5 h_	111.94 ± 116.62	
*VAS focused* (mm)	E_max_	55.22 ± 36.84	**a, b, c**, d
	T_max_ ^a^	0.66 (0.33−1.50)	
	AUC_0−5 h_	111.94 ± 116.62	
*VAS change in colors (mm)*	E_max_ T_max_ ^a^	1.11 ± 2.98 0.0 (0.00−1.00)	NS
	AUC_0−5 h_	1.11 ± 2.98	
*VAS changes in lights* (mm)	E_max_	3.44 ± 8.05	NS
	T_max_ ^a^	0.0 (0.00−1.00)	
	AUC_0−5 h_	3.56 ± 8.11	
*VAS changes in hearing* (mm)	E_max_	1.44 ± 3.64	NS
	T_max_ ^a^	0.0 (0.00−1.00)	
	AUC_0−5 h_	1.56 ± 3.97	
*VAS hallucinations- hearing of sounds or voices* (mm)	E_max_	1.00 ± 3.00	NS
	T_max_ ^a^	0.0 (0.00−1.00)	
	AUC_0−5 h_	1.67 ± 5.00	
*VAS drowsiness* (mm)	E_max_	20.00 ± 25.77	NS
	T_max_ ^a^	2.0 (0.0−5.0)	
	AUC_0−5 h_	33.67 ± 43.75	
VAS dizziness (mm)	E_max_	0.33 ± 1.00	NS
	T_max_ ^a^	0.0 (0.0−1.0)	
	AUC_0−5 h_	0.33 ± 1.00	
VAS confusion (mm)	E_max_	5.56 ± 14.55	NS
	T_max_ ^a^	0.0 (0.0−1.0)	
	AUC_0−5 h_	10.33 ± 28.82	
*VAS different body feeling* (mm)	E_max_	27.44 ± 33.22	**c**
	T_max_ ^a^	1.0 (0.0−2.0)	
	AUC_0−5 h_	43.00 ± 66.32	
*VAS unreal body feeling* (mm)	E_max_	0.22 ± 0.67	NS
	T_max_^a^	0.0 (0.0−1.0)	
	AUC_0−5 h_	0.44±1.33	
*VAS open to others* (mm)	E_max_	53.11 ± 32.64	**a, b, c, d,** e
	T_max_^a^	0.66 (0.33−1.50)	
	AUC_0−5 h_	106.72 ± 102.81	
*VAS trust to others* (mm)	E_max_	54.44 ± 35.79	**a, b, c, d**
	T_max_ ^a^	0.66 (0.0−1.50)	
	AUC_0−5 h_	108.3 ± 100.82	
*VAS feeling close to others* (mm)	E_max_	53.11 ± 34.12	**a, b, c, d**, e
	T_max_^a^	0.66 (0.33−1.50)	
	AUC_0−5 h_	107.02 ± 91.39	
*VAS would like to be with other people* (mm)	E_max_	45.33 ± 34.19	**a, b,** c, d
	T_max_^a^	0.33 (0.00−1.50)	
	AUC_0−5 h_	83.60 ± 77.08	
*VAS would like to hug someone* (mm)	E_max_	29.00 ± 35.13	a, b, c, d
	T_max_ ^a^	0.66 (0.00−1.50)	
	AUC_0−5 h_	64.19 ± 94.79	
*VAS palpitations* (mm)	E_max_	28.00 ± 36.11	a, b
	T_max_ ^a^	0.33 (0.00−1.00)	
	AUC_0−5 h_	43.57 ± 77.98	
*VAS anxiety* (mm)	E_max_	21.22 ± 35.53	NS
	T_max_ ^a^	0.33 (0.00−2.00)	
	AUC_0−5 h_	51.51 ± 94.62	
*VAS sexual desire* (mm)	E_max_	22.11 ± 36.04	NS
	T_max_ ^a^	1.0 (1.0−2.0)	
	AUC_0−5 h_	45.78 ± 87.04	
*VAS sexual arousal* (mm)	E_max_	19.00 ± 37.21	NS
	T_max_ ^a^	0.00 (1.0−2.0)	
	AUC_0−5 h_	43.89 ± 89.86	
ARCI PCAG (score)	E_max_	2.44 ± 4.48	NS
	T_max_ ^a^	2.00 (1.0−5.0)	
	AUC_0−5 h_	2.67 ± 10.42	
ARCI MBG (score)	E_max_	7.78 ± 3.67	**b, c, d**, e, f
	T_max_ ^a^	0.66 (0.66−3.00)	
	AUC_0−5 h_	15.90 ± 10.31	
ARCI LSD (score)	E_max_	1.00 ± 3.84	NS
	T_max_ ^a^	1.0 (1.0−2.0)	
	AUC_0−5 h_	0.61 ± 7.57	
ARCI BG (score)	E_max_	3.78 ± 3.03	**c**
	T_max_ ^a^	1.00 (1.00−3.00)	
	AUC_0−5 h_	7.94 ± 5.50	
ARCI A (score)	E_max_	4.67 ± 1.87	**c**, e, g
	T_max_ ^a^	1.00 (1.00−3.00)	
	AUC_0−5 h_	10.22 ± 5.68	
VESSPA S (score)	E_max_	0.56 ± 0.68	NS
	T_max_ ^a^	1.0 (1.0−4.0)	
	AUC_0−5 h_	1.35 ± 1.80	
VESSPA ANX (score)	E_max_	1.20 ± 0.88	**c, e**
	T_max_ ^a^	1.00 (1.00−2.00)	
	AUC_0−5 h_	2.85 ± 2.18	
VESSPA CP (score)	E_max_	0.02 ± 0.06	NS
	T_max_ ^a^	0.00 (0.00−1.00)	
	AUC_0−5 h_	0.02 ± 0.06	
VESSPA SOC (score)	E_max_	1.31 ± 0.77	**c, e**
	T_max_ ^a^	1.00 (1.00−5.00)	
	AUC_0−5 h_	2.51 ± 1.94	
VESSPA ACT (score)	E_max_	1.41 ± 0.78	**c, e**
	T_max_ ^a^	1.0 (1.00−5.00)	
	AUC_0−5 h_	2.57 ± 1.44	
VESSPA PS (score)	E_max_	0.37 ± 0.39	**c, e**
	T_max_ ^a^	1.0 (1.00−2.00)	
	AUC_0−5 h_	0.80 ± 1.02	

Emax, peak effects 0−5 h (differences from baseline) measured by mm (visual analog scale [VAS]), and score (Addiction Research Center Inventory [ARCI], Evaluation of Subjective Effects of Substances with Abuse Potential questionnaire [VESSPA-SEE]) and expressed as mean ± standard deviation. A post-hoc Dunnett’s test for multiple comparisons was used. Statistical differences are presented as “a” p < 0.05, “**a**” p < 0.01 (times 0− 0.33 h), “b” p < 0.05, “**b**” p < 0.01 (times 0− 0.66 h), “c” p < 0.05, “**c**” p < 0.01 (times 0− 1 h), “d” p < 0.05, “**d**” p < 0.01 (times 0− 1.5 h), “e” p < 0.05, “**e**” p < 0.01 (times 0−2 h) and “f” p < 0.05, “**f**” p < 0.01 (times 0−2.5 h), “g” p < 0.05 “**g**” p < 0.01 (times 0− 3 h), “h” p < 0.05, “**h**” p < 0.01 (times 0− 4 h) and “i” p < 0.05, “**i**” p < 0.01 (times 0− 5 h). NS, not significant. VASs in italics are measured 0, 0.33, 0.66, 1, 1.5, 2, 2.5, 3, 4, 5 h. The other VASs, ARCI_PCAG/LSD/BG/A and VESSPA at 0, 1, 2, 3, 4, 5 h, and ARCI MBG at 0, 0.66, 1, 1.5, 2, 2.5, 3, 4, 5 h.

a For T_max_ data are reported, as median and range.

Compared to the baseline, the highest scores (a difference of >50 mm from baseline) in Visual Analog Scales (VASs) were observed for liking the effect, clarity, being focused, openness to others, trust in others, and closeness to others. Differences of >25 mm from baseline were obtained for intensity, stimulation, high, good effects, enjoyment being with other people, desire to hug someone, palpitations, and different body feeling. Moderate (10–15 mm) and small changes (<10 mm) showed no significant differences from baseline, in the scales measuring bad effects, anxiety, changes in colors, changes in lights, changes in hearing, hallucinations-hearings of sounds or voices, drowsiness, dizziness, confusion, unreal body feeling, sexual desire and sexual excitement. There were no significant differences from baseline in changes related to distance, shapes, light or spot hallucinations, animal hallucinations, objects, insects, or people, as well as perceptions of different or unreal surroundings. In the Addiction Research Center Inventory questionnaire (ARCI), significant differences from baseline were found in the MBG (morphine-benzedrine group, euphoria), BG (benzedrine group, intellectual efficiency and energy), and A (amphetamine-like effects) subscales.

Regarding the Evaluation of Subjective Effects of Substances with Abuse Potential (VESSPA-SSE), α-PVP caused significant changes compared to baseline in several subscales, such as ANX (anxiety), SOC (pleasure and sociability), ACT (activity and energy), and PS (psychotic symptoms).

**FIGURE 2:**
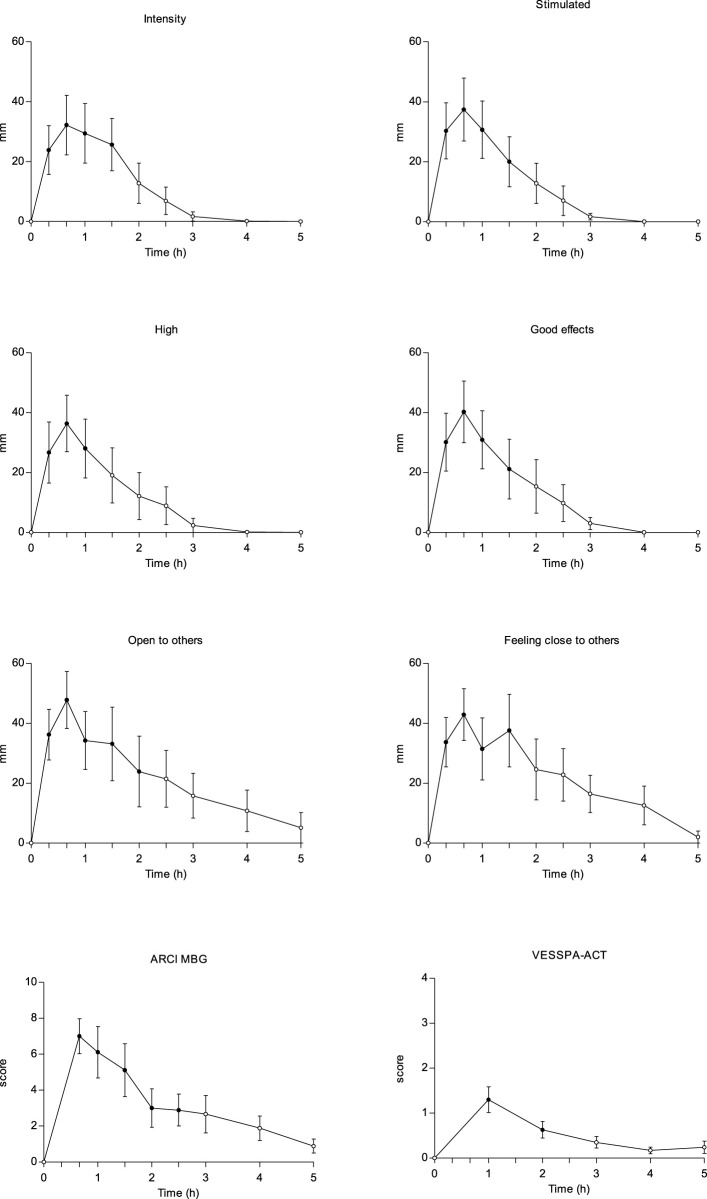
Time course (n = 9; mean ± standard error) of subjective effects following intranasal administration of 10–20 mg α-PVP. Significant differences from the baseline are indicated with filled symbols • (p < 0.05). Includes intensity, stimulation, high, good effects, openness to others, feeling close to others, ARCI MBG and VESSPA-ACT. ARCI (Addiction Research Center Inventory questionnaire) subscale MBG (morphine-benzedrine group, euphoria) and VESSPA (Evaluation of Subjective Effects of Substances with Abuse Potential) subscale ACT (activity and energy). Additional outcomes are shown in Supplementary Figure S1.

Figure 2 shows the main effects including intensity, stimulation, high, good effects, openness to others, feeling close to others, ARCI MBG, and VESSPA-AE. Additional outcomes are shown in Supplementary Figure S1.

### 3.4 Adverse effects

No serious adverse effects were reported. One of the nine subjects experienced a mild headache 5 h after administration, which resolved with a non-steroidal anti-inflammatory drug (600 mg of ibuprofen). No changes were observed in the Positive and Negative Syndrome Scale for Schizophrenia (PANNS) score.

## 4 Discussion

To the best of our knowledge, this is the first observational study under naturalistic conditions to investigate the acute pharmacological effects of intranasal α-PVP in humans, providing unique insights into its acute subjective and physiological effects. Our primary findings demonstrate that α-PVP exhibits characteristic psychostimulant effects, including pronounced cardiovascular responses such as increased heart rate, systolic blood pressure, and diastolic blood pressure. Additionally, α-PVP produced notable subjective effects, including feelings of wellbeing, stimulation, euphoria, openness to others, closeness to others, enjoyment of the effect, clarity, and focus. These results align with empathogenic and psychostimulant effects and are comparable to those observed after consuming other substances, such as MDMA, mephedrone, and methylone, which are primarily empathogenic, as well as substances with stronger psychostimulant effects, such as cocaine and amphetamines (Papaseit et al., 2016; Poyatos et al., 2023). Hallucinogenic effects were not observed, and no changes in shapes, distances, or lights were reported. The understanding of α-PVP-related symptoms is of primary importance for the individuation of the possible cause of the intoxications, which may support the toxicologists to conduct specific analysis. α-PVP related acute intoxications resulted in the fatality of an obese subject without previous history of drug abuse (Nóbrega and Dinis-Oliveira, 2018). Furthermore, α-PVP was reported in drive under the influence of drug-case in which the subject presented different symptoms, typical of synthetic cathinones’-related intoxication. In this case, the toxicological analyses confirmed the consumption α-PVP (Ellefsen et al., 2016). A similar observational study was performed with methylone by oral route, where participants self-administered orally 100–300 mg and increased heart rate and typical stimulant and empathogenic effects were observed (Poyatos et al., 2021; Papaseit et al., 2021). This study also compared the effects of methylone with MDMA oral self-administration (doses from 75 to 100 mg), the last one showing similar but less intense physiological and subjective effects. Analogously, a clinical trial comparing the effects of 200 mg methylone with 100 mg MDMA and placebo by oral route, demonstrated comparable physiological effects and subjective effects of both drugs. Methylone showed a faster overall onset and earlier disappearance of subjective effects in comparison to those associated with MDMA (Poyatos et al., 2023).

In the case of mephedrone, 10 experienced drug users self-administered mephedrone 100–200 mg orally or 50–100 mg intranasally. Results showed an increase in both groups on systolic and diastolic blood pressure, temperature and heart rate without significant differences among the routes of administration in vital signs except for cutaneous temperature (Emax). One limitation of the study was that the first assessment occurred 1 hour after self-administration, potentially missing the peak effects for some outcomes (Poyatos et al., 2021).

A recently published crossover, placebo-controlled trial investigated the effects of oral administration of 3-methylmethcathinone (3-MMC) at doses of 25, 50, and 100 mg. Participants in that study reported mild increases in dissociative and psychedelic effects, which were not observed after consuming α-PVP. Additionally, sympathomimetic effects previously described in this class of substances were observed, resembling those of MDMA and amphetamines (Ramaekers et al., 2024).

In this study, the physiological and subjective effects of α-PVP were quite similar to those reported for mephedrone, methylone and 3-MMC, but with greater intensity (Papaseit et al., 2016; Poyatos et al., 2023; Ramaekers et al., 2024). Another difference is the earlier presentation of effects and oral concentrations with α-PVP (also shorter Tmax values) likely due to the intranasal administration of α-PVP compared to the oral administration of methylone and mephedrone, as well as the different time points of assessment.

For α-PVP, the intensity and stimulated effects peaked at 0.66 h when administered intranasally. In the case of orally taken mephedrone (200 mg) and methylone (200 mg), the peak effects were observed at 0.75 h.

It was not possible to calculate the elimination half-life of α-PVP but oral fluid concentrations were lower after 5 h in comparison to MDMA or methylone. All the participants had concentrations of α-PVP in oral fluid, with a peak within the first hour and lasting until the end of the session (Di Trana et al., 2025). According to these preliminary results, oral fluid could be a suitable biological matrix to detect recent α-PVP use. Additionally, α-PVP concentrations in urine were higher from 2 to 5 h of administration and ten possible metabolites were identified (Di Trana et al., 2025).

The variables intensity and stimulation exhibited a strong correlation between subjective effects and oral fluid concentration, with correlation coefficients of 0.71 (p-value = 0.045) and 0.76 (p-value = 0.028), respectively. The peak subjective effect occurred within the first hour, while the peak concentration reached 0.33 h.

It should be noticed that nor α-PVP, mephedrone, methylone or MDMA induced hallucinations, psychotic episodes, or any other serious adverse events during the experimental or naturalistic sessions. The explanation can be that these effects are reported with higher doses (in cases of intoxication) than those administered in reported studies (low or moderate).

Regarding MDPV, potent stimulant effects have been described due to its action as a dopamine and norepinephrine reuptake inhibitor (Baumann et al., 2017). So far, no human experimental studies on MDPV have been conducted. Surveys and cases of intoxication have reported tachycardia, hypertension, hyperthermia, mydriasis, and muscle tension; effects that are similar to those of α-PVP. However, MDPV is more potent than both α-PVP and cocaine (Froberg et al., 2015). Additionally, MDPV induces euphoria, heightened alertness and energy, but also anxiety, paranoia, and a strong craving for re-consumption, with more intense and compulsive characteristics compared to α-PVP, which has a similar profile but with shorter duration and reduced intensity. Moreover, MDPV does not produce empathogenic effects, setting it apart from cathinones such as methylone, and primarily categorizing it as a psychostimulant drug (Karila et al., 2018; Desharnais et al., 2017).

A systematic review assessed the pharmacological effects related to the abuse potential and pharmacokinetics of cathinones (Poyatos et al., 2022a). It described increased blood pressure, heart rate, and a subjective euphoric effect characterized by heightened energy and motor stimulation. The cathinones studied include methylone, mephedrone, cathinone and diethylpropion. Mephedrone and methylone primarily exhibited empathogenic effects, while pyrrolidine derivatives like MDPV mainly displayed psychostimulant effects, according to mechanistic studies (Baumann et al., 2017; Baumann et al., 2018).

Previous studies on other psychostimulants, particularly cocaine, indicate that an increase in heart rate and blood pressure are key physiological effects. Well-known effects of cocaine include euphoria, enhanced awareness, heightened alertness, and a diminished need for sleep (Farré et al., 1997). For methamphetamine, also sympathomimetic effects have been reported after intranasal administration (dose range 5–30 mg) like increased blood pressure, heart rate, and body temperature. However, following α-PVP administration, no statistically significant temperature increase was observed. Subjective effects included good feelings, liking the substance, and cravings for re-consumption, with magnitudes comparable to prior studies (Rush et al., 2011; Reynolds et al., 2017). Cathinones like α-PVP therefore produce similar effects of other psychostimulants (Poyatos et al., 2022b).

The limitations of this study should be acknowledged. The observational design inherently reflects typical limitations, such as potential selection bias towards participants with extensive experience in substance use, limiting its generalization to light consumers. Also, no blood samples were collected since this observational study was conducted in a naturalistic setting. Additional limitations include a relatively small sample size and the absence of direct control or comparisons with other substances or placebo.

The limited sample size and poor gender representation limits the identification of dose- or gender-specific effects. Also, it could have influenced the results, particularly when applying Dunnett’s post hoc test, which may not have been sufficiently powered to detect significant differences in this context. Future studies could benefit from *a priori* sample size calculation to ensure adequate statistical power for these comparisons.

The intense monitoring during the study could have resulted in stress-related effects. However, at the end of the sessions, participants were asked to define the sensations they felt in their own words and none of them described the experience as stressful. Two participants would have liked fewer interruptions. Measurement of salivary cortisol could be useful in addressing this issue in future studies.

The gold standard to evaluate subjective effects of substances is a randomized double-blind, placebo-controlled study, although previous drug use/drug experience of participants can affect the validity of the blinding group due to the expectations of the effects based on previous consumption experience. We have previously evaluated participants with previous experience in psychostimulant consumption in two published double-blind and placebo-controlled clinical trials with MDMA, mephedrone and methylone (Papaseit et al., 2016; Poyatos et al., 2023). The results showed that between 92% and 94% of subjects correctly recognized when they had received a placebo. Furthermore, between 83% and 94% of participants identified correctly the administered substance.

The subjective and physiological effects observed in other naturalistic and observational studies with psychostimulants and some psychedelics were very similar to those observed previously in double blind placebo-controlled studies. This overlap in the profile of pharmacological effects (subjective and physiological) has been documented for MDMA (Papaseit et al., 2016; Khan et al., 2013; Poyatos et al., 2023; Angerer et al., 2024; Irvine et al., 2006; Morefield et al., 2011), mephedrone (Papaseit et al., 2016; Papaseit et al., 2021; Papaseit et al., 2020b; Freeman et al., 2012), methylone (Poyatos et al., 2023; Poyatos et al., 2021; Poyatos et al., 2022b), 4-bromo-2,5-dimethoxyphenethylamine (2C-B, Nexus) (Mallaroni et al., 2023; Papaseit et al., 2018), and 5-methoxy-N,N-dimethyltryptamine (5-MeO-DMT, mebufotenin (Papaseit et al., 2018; Timmermann et al., 2025). The main effects were comparable in both methodological approaches, and only some variations in the intensity and magnitude of subjective responses were detected, especially when considering the different doses used. These findings reinforce the validity of observational studies conducted under standardized conditions, and the results presented here.

## 5 Conclusion

This observational study constitutes an initial approach to assess the acute physiological and subjective pharmacological effects of the intranasal administration of known doses of α-PVP in humans. Results suggest that intranasal α-PVP self-administration in experienced drug users, in a non-controlled setting, induces a constellation of psychostimulant-like effects, but also empathogen effects commonly associated with drugs like MDMA and other cathinones like mephedrone and methylone.

Despite its limitations, this research underscores the need for further investigations with other psychostimulants under controlled conditions and mechanistic studies to deepen understanding of its pharmacological profile and abuse potential in humans. Additionally, gender differences should be addressed in future studies.

## Data Availability

The raw data supporting the conclusions of this article will be made available by the authors, without undue reservation.
