# Supplementary material for: Acute pharmacological effects of α-PVP in humans: a naturalistic observational study
Source: Front Pharmacol. 2025 Jul 2;16:1626692. doi: 10.3389/fphar.2025.1626692 (PMC12263604; doi:10.3389/fphar.2025.1626692)
Supplement: Supplementary file 1 [file Image1.pdf]

Supplementary Material

1 Supplementary Figure

Supplementary Figure 1 shows the time course of other subjective effects.

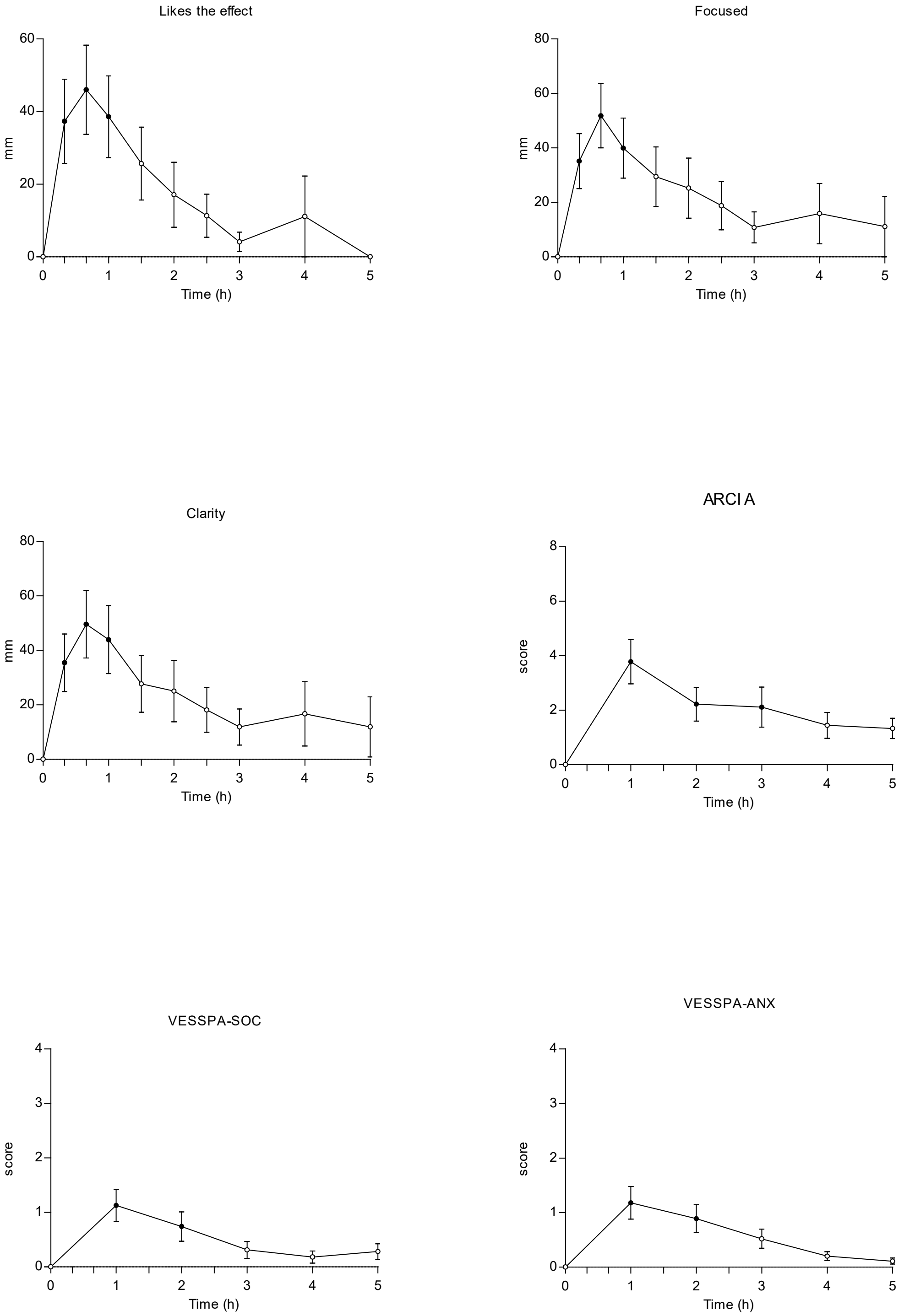

Supplementary Figure 1. Time course (n=9; mean ± standard error) of subjective effects following intranasal administration of 10-20mg α-PVP. Significant differences from the baseline are indicated with filled symbols ● ($p < 0.05$). ARCI (Addiction Research Center Inventory questionnaire) subscale A (amphetamine-like effects). VESSPA (Evaluation of Subjective Effects of Substances with Abuse Potential) subscale ANX (anxiety) and subscale SOC (pleasure and sociability).
